# In vitro dry matter and crude protein rumen degradation and abomasal digestibility of soybean meal treated with chestnut and quebracho wood extracts

**DOI:** 10.1002/fsn3.2072

**Published:** 2020-12-22

**Authors:** Andrej Lavrenčič, Alenka Levart

**Affiliations:** ^1^ Department of Animal Science Biotechnical Faculty University of Ljubljana Domžale Slovenia

**Keywords:** by‐pass protein, chestnut wood extract, in vitro degradability, in vitro digestibility, quebracho wood extract, rumen

## Abstract

The effects of commercial chestnut (CWE) and quebracho (QUE) extract at different inclusion levels to soybean meal (SBM) on the in vitro degradability and digestibility of dry matter (DM) and crude protein (CP) were evaluated. Samples were prepared by mixing 0 (CON), 15, 30, and 60 g/kg of CWE and QUE with SBM, soaked in water overnight at room temperature, dried, and ground. Samples were incubated in duplicate in buffered rumen fluid for 24 hr at 39°C. In vitro rumen degradability of DM and CP of tannin‐treated SBM decreased with increasing quantities of tannins, especially with CWE‐treated SBM. In vitro abomasal (pepsin‐HCl) digestibility of the DM and CP was only slightly suppressed. As a result, rumen by‐pass protein (BP‐CP) increased with increased quantities of tannins, especially with CWE‐treated SBM. In comparison with nontreated SBM, the BP‐CP digestibility did not decrease, except with the highest quantity of QUE. Treatment of the SBM with tannins, especially with CWE, increased flow of the undegraded protein to the abomasum, suggesting the better supply of the ruminant animal with amino acids.

## Graphical abstract

In vitro degradabilities and digestibilities of dry matter and crude protein were determined in soybean meal treated with quebracho and chestnut tannin extracts. Chestnut tannins decreased the rumen crude protein degradability to a greater extent than quebracho tannins thus providing a greater amount of by‐pass protein.

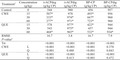

## 1 INTRODUCTION

During digestive processes in the rumen, proteins are partially or wholly degraded by digestive flora present in the rumen. The rumen degradation of plant proteins often exceeds 60% (AFRC, 1993). In the last decades, treatments of proteinaceous feeds with bioactive phytochemicals, predominantly tannins, to decrease the excessive degradation of nutrients in the rumen gained a lot of attention. Tannins are the plant secondary compounds, mainly polyphenols, which can form complexes with proteins, carbohydrates, and other substances present in the feed (McSweeney et al., 2001; Mueller‐Harvey, 2006). Free tannins or tannin‐protein complexes may alter the susceptibility of the feed protein to bacterial action and impair the activity of proteolytic enzymes in the digestive tract. The formation of complexes depends on the type and/or concentration of tannins and on type of proteins (Dawson et al., 1999; Mueller‐Harvey, 2006). In case that tannin‐protein complexes escape bacterial degradation in the rumen and are dissociated and proteolyzed in the lower part of the digestive tract, the released amino acids can be absorbed by the host animal. The most frequently used tannins in ruminant nutrition are water extracts from chestnut (CWE) and quebracho (QUE) wood, in which hydrolyzable and condensed tannins prevail, respectively. Both types of tannins are given to animals first of all to decrease the rumen degradability of dietary protein, but also to prevent bloat, to mitigate rumen methane emissions, to inhibit the biohydrogenation of polyunsaturated fatty acids in the rumen and to decrease the infestations by gastro‐intestinal parasitic nematodes (Aguerre et al., 2016; Beauchemin et al., 2007; Buccioni et al., 2015; Niezen et al., 1995; Waghorn et al., 1987).

Generally, the CWE and QUE are simply added to the proteinaceous feeds, such as soybean meal (SBM). However, such supplementation does not always give consistent results, most probably because the concentrations of CWE or QUE are too low or because the tannins are not forming complexes with proteins in a great extent. We hypothesized that the complexation at low concentrations of tannins will be more efficient if feeds will be treated by soaking the mixture of tannin and feed in the water as suggested by Driedger and Hatfield (1972). The objective of this research was to determine the in vitro rumen degradability and abomasal digestibility of dry matter and crude protein in SBM, treated by soaking it with chestnut wood (CWE) and quebracho wood (QUE) water extracts.

## 2 MATERIAL AND METHODS

### 2.1 Soybean meal, tannin sources, and preparation of soybean meal‐tannin mixtures

Soybean meal and commercial dry water extracts of sweet chestnut (CWE; *Castanea sativa* L.) and quebracho wood (QUE; *Shinopsis* spp.) were used. Both extracts were obtained from a company Tanin Sevnica (Sevnica, Slovenia). SBM (500 g) was weighed in 3 L containers and 0 (control), 15, 30, and 60 g/kg SBM of one of the tannin sources (e.g., CWE or QUE) were added in powder form. The concentrations varied between those recommended by manufacturer or dealer (around 20 g/kg DM) and those at which the tannins should have negative effects on digestion (>50 g/kg DM). The SBM and tannin extracts were homogenized by mixing manually until an even color is obtained. Tap water (app. 2,500 g/kg mixture) was added progressively to completely cover the mixture and the mass was stirred manually until a homogenous brownish paste was obtained. The mixture was then left to stand for 12 hr (overnight) at room temperature, dried, ground into fine particles (app. 1 mm) and stored in dark at ambient temperature until analyzed. All substrate preparation for each tannin treatment and the control (CON; soaked with water without tannin extract) were made in duplicate. The composition of substrates is presented in Table 1.

**TABLE 1:** Chemical composition of soybean meal (g/kg DM) treated with chestnut (CWE) and quebracho (QUE) wood extracts

	Treatment^a^						
	CON	CWE			QUE		
		15	30	60	15	30	60
DM (g/kg)	923	911	867	851	912	933	920
CP	567	524	525	501	522	533	524
EE	11	14	14	14	11	12	13
CF	58	49	51	47	48	51	50
Ash	78	76	74	72	75	74	76
NFE	285	337	336	366	344	331	338
NDF	111	102	112	104	104	133	121
NFC	233	284	275	309	288	248	266

Note

Abbreviations: CON, control; CWE, chestnut wood extract; QUE, quebracho wood extract; DM, dry matter; CP, crude protein; EE, ether extract; CF, crude fiber; Ash, crude ash; NFE, nitrogen‐free extract; NDF, neutral detergent fiber; NFC, non‐fiber carbohydrates (1,000−[CP + EE + Ash + NDF]);

^a^ Both extracts were added at 15, 30 and 60 g/kg of substrate.

### 2.2 In vitro degradability and digestibility

Sheep rumen fluid was derived from two mature castrated Jezersko Solčavska × Romanovska rams (*Ovis aries*), weighing on average 80 kg and fitted with permanent rumen cannula. They received a daily ration containing average quality hay fed ad libitum and 0.25 kg of commercial compound feed (180 g/kg crude protein [CP]), supplemented with mineral and vitamin mix (0.025 kg) once a day. The diet composition was calculated according to the German metabolizable energy (ME) and utilizable protein requirements (nXP; DLG Futterwerttabellen: Wiederkäuer, 1997) to cover the energy and protein requirements for maintenance and to balance the energy‐to‐protein ratio in the rumen. Animals were kept following animal welfare regulation (U33401‐12/2019/9 from 16.7.2019 issued by Food Safety, Veterinary and Phytosanitary Inspection, Ministry of Agriculture, Forestry and Food, Administration of the Republic of Slovenia for Food Safety, Veterinary and Plant protection, Ljubljana, Slovenia).

In vitro incubations were carried out by using ANKOM in vitro fermentation system Daisy^II^ (ANKOM Technology). Inocula was prepared according to Menke and Steingass (1988). In the laboratory, sheep rumen fluid was strained through four layers of cheesecloth. Obtained rumen fluid was diluted using the reduced buffer medium in the proportion 1:4 (v/v), according to ANKOM manual (ANKOM Technology). Approximately four grams of ground air dry substrates were weighed into ANKOM 510 nylon bag (ANKOM Technology), heat‐sealed and placed in incubation vessels. Each substrate was weighed into eight bags, which were placed into four vessels (2 bags/sample per vessel). Each vessel contained 16 bags (2 bags/substrate + two blanks). Two liters of buffered rumen fluid, dispensed under CO_2_, were poured into each vessel, which was then deposited into the rotating incubator at 39°C for 24 hr. After the incubation, all bags (*n* = 56) were rinsed thoroughly with cold tap water until the water was clear. Half of the bags (*n* = 28) were dried at 48°C for at least 48 hr, weighed and in vitro apparent dry matter (DM) degradabilities (ivADMdeg) were calculated. Remaining bags (*n* = 28) were treated immediately after rinsing with water with pepsin–HCl solution (abomasal digestibility; Tilley & Terry, 1963) for 24 hr. After the end of this treatment, bags were rinsed thoroughly with cold tap water until the water was clear, dried at 48°C for at least 48 hr, weighed and in vitro apparent DM digestibilities (*iv*ADMdig) were calculated. All incubations were performed in two consecutive weeks, each week representing one run.

After incubations, the remaining material from bags was pooled according to the container (first or second), in vitro determination (degradability or digestibility), type of tannin (CON, CWE or QUE), the concentration of tannin (15, 30, or 60 g/kg SBM), and consecutive run (first or second). Altogether 28 samples were obtained and in each sample, the CP content was determined according to Naumann and Bassler (1976) in all samples, while the neutral detergent fiber (NDF) content (Goering & Van Soest, 1970) was determined in 14 samples after in vitro rumen incubation. From these data in vitro apparent CP degradabilities (*iv*ACPdeg), in vitro apparent CP digestibilities (*iv*ACPdig) and in vitro true DM digestibilities (*iv*TDMdig; Van Soest, 1994) were calculated. The sum of the difference between *iv*ACPdig and *iv*ACPdeg and nondigestible CPs gave the by‐pass CP (BP‐CP) content. BP‐CP was then multiplied with *iv*ACPdig to estimate the digestibility of BP‐CP (BP‐CPdig; Cortés et al., 2009).

### 2.3 Statistical analyses

In the experiment, the fermentation runs were performed in different periods (weeks) and replicates between runs were the statistical replicates. Data were analyzed with the mixed model procedure (Statistical Analysis Systems Institute, 1994). Tannin treatment means for each level were compared against the CON using the least square mean linear hypothesis (LSMEANS/DIFF) with the Dunnett adjustment. Orthogonal polynomial contrasts were adjusted to the unequally spaced levels to determine linear and quadratic responses to the level of tannin (0, 15, 30, and 60 g of tannin/kg of SBM) within each tannin source.

## 3 RESULTS

The values of *iv*ADMdeg, *iv*TDMdig, and *iv*ADMdig are presented in Table 2.

**TABLE 2:** Effects of chestnut wood and quebracho wood extracts on *iv*ADMdeg, *iv*TDMdig, and *iv*ADMdig over a 24‐hr rumen incubation period

Treatment^a^	Concentration (g/kg)	*iv*ADMdeg (g/kg DM)	*iv*ATDMdig (g/kg DM)	*iv*ADMdig (g/kg DM)
Control	0	639	859	948
CWE	15	636	759*	953
	30	537*	704*	946
	60	515*	689*	938
QUE	15	682*	862	950
	30	657	848	947
	60	606*	841	937
RMSE		12.3	27.3	7.9
*p*‐value^b^				
Trt		<0.001	<0.001	0.078
CWE	L	<0.001	<0.001	0.014
	Q	0.007	<0.001	0.282
QUE	L	<0.001	0.226	0.016
	Q	<0.001	0,938	0.261

Note

Abbreviations: CON, control; CWE, chestnut wood extract; QUE, quebracho wood extract; *iv*ADMdeg, *in vitro* apparent DM degradability; *iv*ADMdig, *in vitro* apparent DM digestibility; *iv*TDMdig, *in vitro* true DM digestibility.

^a^ Both extracts were added at 15, 30 and 60 g/kg of substrate.

^b^ *p*‐values for Trt = treatments; orthogonal contrasts for L = linear and Q = quadratic effects.

* Means within a column differ significantly from the CON (*p* < .05).

The *iv*ADMdeg were affected by experimental treatments (*p* < .001), showing linear and quadratic response for both tannin extracts (*p* < .001). The lowest dosage of CWE (15 g/kg SBM) and middle dosage (30 g/kg SBM) of QUE did not affect the *iv*ADMdeg, while the lowest dosage of QUE even increased the *iv*ADMdeg by about 7% (*p* < .05). The dosage of 30 g CWE and 60 g/kg of CWE or QUE decreased (*p* < .05) the *iv*ADMdeg by about 16, 19, and 5%, respectively. The *iv*TDMdig were affected by experimental treatments (*p* < .001), showing linear and quadratic response only when SBM was treated with CWE (*p* < .001). The reductions in *iv*TDMdig of about 12, 18, and 20% were observed when SBM was treated with 15, 30, and 60 g of CWE, respectively. The experimental treatment only tended to effect *iv*ADMdig (*p* = .078) and both tannin extracts showed only a linear response on the *iv*ADMdig (*p* < .05).

The effects of SBM treated with increasing amounts of tannin extracts on *iv*ACPdeg, *iv*ACPdig, BP‐CP, and BP‐CPdig are presented in Table 3.

**TABLE 3:** Effects of chestnut wood and quebracho wood extracts on *iv*ACPdeg and *iv*ACPdig and BP‐CP contents and BP‐CPdig (g/kg CP) over a 24‐hr incubation period

Treatment^a^	Concentration (g/kg)	*iv*ACPdeg (g/kg CP)	*iv*ACPdig (g/kg CP)	BP‐CP (g/kg CP)	BP‐CPdig (g/kg CP)
Control	0	544	980	456	957
CWE	15	507*	978	493*	956
	30	333*	974*	667*	960
	60	277*	971*	723*	960
QUE	15	578	977*	422*	944
	30	543	975*	457	944
	60	468*	965*	532*	934*
RMSE		16.7	3.8	16.7	7.9
*p*‐value^b^					
Trt		<0.001	<0.001	<0.001	<0.001
CWE	L	<0.001	<0.001	<0.001	0.370
	Q	<0.001	0.488	<0.001	0.843
QUE	L	<0.001	<0.001	<0.001	<0.001
	Q	<0.001	0.413	<0.001	0.472

Note

Abbreviations: CON, control; CWE, chestnut wood extract; QUE, quebracho wood extract; *iv*ACPdeg, *in vitro* apparent CP degradability; *iv*ACPdig, *in vitro* apparent CP digestibility; BP‐CP, CP undegraded with ruminal fluid (by‐pass CP); BP‐CPdig, digestibility of by‐pass CP in pepsin‐HCl.

^a^ Both extracts were added at 15, 30 and 60 g/kg of substrate.

^b^ *p*‐values for Trt = treatments; orthogonal contrasts for L = linear and Q = quadratic effects.

* Means within a column differ significantly from the CON (*p* < .05).

The experimental treatment affected all studied parameters (*p* < .001). The *iv*ACPdeg and BP‐CP showed linear and quadratic responses (*p* < .001) when SBM was treated with both tannin extracts. *Iv*ACPdig showed linear responses (*p* < .001) when SBM was treated with both tannin extracts, while BP‐CPdig showed a linear response only with QUE treatment (*p* < .001). The *iv*ACPdeg decreased (*p* < .05) already when SBM was treated with 15 g of CWE (decrease of about 7%). SBM treated with 30 and 60 g of CWE further decreased *iv*ACPdeg by about 39 and 49%, respectively. On contrary, only the treatment of SBM with 60 g of QUE reduced *iv*ACPdeg by about 14% (*p* < .05). The treatment of SBM with CWE and QUE reduced (*p* < .001) also the *iv*ACPdig. However, the reduction in *iv*ACPdig was small, being 0.2, 0.6, and 0.9% when SBM was treated with 15, 30, and 60 g of CWE, respectively, and 0.3, 0.5, and 1.5% when SBM was treated with 15, 30, and 60 g of QUE, respectively. Treatment with 15, 30, and 60 g of CWE increased the content of BP‐CP by 8, 46, and 59%, respectively. On contrary, treatment of SBM with 15, 30, and 60 g of QUE decreased (by 7%) had no effect and increased (by 17%) the BP‐CP content of SBM. The treatments of SBM with CWE and QUE did not affect the BP‐CPdig (*p* > .05), except the treatment with 60 g of QUE which decreased the BP‐CPdig by about 2.5% (*p* < .05).

## 4 DISCUSSION

The treatment of SBM with increasing amounts of CWE and QUE decreased the *iv*ADMdeg (Table 2). This decrease was more prominent when SBM was treated with CWE than with QUE and this is in close agreement with the results of Gonzáles et al. (2002) but not with the results of Driedger and Hatfield (1972), Frutos et al. (2000), and Hervás et al. (2000), who reported a greater effect of QUE. Like *iv*ADMdeg also the *iv*ACPdeg (Table 3) was more affected by CWE than by QUE, which is in agreement with the results of Gonzáles et al. (2002) and Kondo (2010). The greater reduction of *iv*ACPdeg in CWE‐treated SBM in comparison with QUE‐treated SBM was presumably caused by the complexation of CWE hydrolyzable tannins with SBM CP. We suppose that during the treatment the hydrolyzable tannin phenolic groups formed bonds with SBM nutrients which are resistant in rumen environment. Dawson et al. (1999) noted that highly astringent (hydrolyzable) tannins possess the greatest affinity for proteins and Jayanegara et al. (2019) demonstrated that hydrolyzable tannins such as CWE had higher protein precipitation capacity and, consequently, biological activity than condensed tannins, such as QUE.

Several authors suggested that complexes between tannins and nutrients dissociate at the pH lower than 3.5 or pH greater than 8.5 (Jones & Mangan, 1977; McLeod, 1974). Therefore, in the rumen the nutrients may be bound and protected from microbial degradation, but are released in the abomasum, enabling their digestion and absorption in the small intestine. These suggestions were proven also in our trial. Treatments of SBM with increasing concentrations of CWE or QUE did not have any effect on *iv*ADMdig (Table 2) and *iv*ACPdig (Table 3), where only the highest concentration of QUE decreased the *iv*ACPdig. Gonzáles et al. (2002) and Cortés et al. (2009) also reported that SBM treated with increasing concentrations of QUE or purified condensed tannins from tropical legumes, respectively, linearly decreased the *iv*ADMdig. According to our results, Frutos et al. (2000), who treated the SBM with QUE, did not found reductions in in vitro CP intestinal digestibility at inclusion levels between 10 and 150 g QUE/kg SBM, while Kondo (2010), who treated SBM with 100 g CWE or QUE/kg SBM, found a decrease in in vitro CP intestinal digestibility of 15 and 27%, respectively. From these results, it could be suggested that the concentrations of CWE and QUE used in our study were too low to decrease the SBM digestibility. This is in agreement with commonly accepted fact, that tannins show antinutritional properties only when they are present in concentrations greater than 50 g/kg DM. The study of Kondo (2010) also showed that treatment with QUE had a more negative effect on CP digestibility than CWE, suggesting that QUE bind on feed constituents more strongly than CWE. This makes the CP unavailable to digestive enzymes in the stomach and intestines (Jayanegara et al., 2019), probably due to different types of bonds formed by CWE and QUE tannins.

Treatment of SBM with increased concentrations of CWE or QUE increased the amount of rumen by‐pass CP (BP‐CP; Table 3). Our results agree with the results of Kondo (2010), who determined that the increase in BP‐CP is higher when SBM was treated with 100 g of CWE than with the same amount of QUE. Cortés et al. (2009) demonstrated that treatment of SBM with high concentrations of isolated tropical legume condensed tannins (from 140 to 420 g/kg DM) also markedly increased the BP‐CP of SBM. A ruminant animal amino acid supply is not dependent only on BP‐CP flow to the abomasum but also on the abomasal digestibility of BP‐CP (BP‐CPdig), which could be affected by the tannins. In our study, the BP‐CPdig was not affected by tannin treatment (up to 2%). It seems that the used concentrations of CWE and QUE were too low, as the BP‐CPdig calculated from the data of Hervás et al. (2000) were lower from 10% to 18% when SBM was treated with 130 and 200 g/kg of tannic acid, while the treatment of SBM with 100 g of CWE even increased the BP‐CPdig by 10% (Kondo, 2010). On the contrary, the treatment of SBM with 100 g of QUE decreased the BP‐CPdig by 50% (Kondo, 2010) or by 10%–37% when SBM was treated with condensed tannins isolated from tropical legumes (Cortés et al., 2009). The BP‐CPdig is thus affected by the type and by the dose of applied/supplemented tannins.

To our knowledge, the *iv*TDMdig of SBM and other feeds treated with CWE and/or QUE has not been previously reported. The CWE reduced the *iv*TDMdig, while QUE did not have any effect (Table 2). This suggests that CWE form the complexes with SBM constituents that are resistant to boiling neutral detergent solution. Thus, determination of DM digestibility by determining *iv*TDMdig (Van Soest, 1994) could be questioned when feeds (e.g., SBM) are treated with tannins. It can be assumed that in tannin‐treated feeds the *iv*TDMdig shows only the potential degradability of DM and not whole‐tract DM digestibility.

## 5 CONCLUSIONS

The results of the present experiment suggest that condensed tannins from quebracho were less effective in protecting SBM from in vitro rumen degradation than hydrolyzable tannins from chestnut, which resulted in a lower quantity of rumen by‐pass protein. This effect is dependent on the type of tannin and its dose used to treat the SBM. Under conditions simulating digestion in the abomasum, the complexes formed either with CWE or QUE almost completely disintegrated. This supports the hypothesis that CWE might be used as a natural additive for improving the digestive utilization of protein‐rich feeds in ruminants.

## CONFLICT OF INTEREST

Authors declare that they do not have any conflict of interests.

## ETHICAL APPROVAL

This study was approved by Food Safety, Veterinary and Phytosanitary Inspection, Ministry of Agriculture, Forestry and Food, Administration of the Republic of Slovenia for Food Safety, Veterinary and Plant protection, Ljubljana, Slovenia. The approval number is U33401‐12/2019/9 issued on 16.7.2019.
